# Accuracy study on “Osteorisk”: a new osteoporosis screening clinical tool for women over 50 years old

**DOI:** 10.1590/S1516-31802008000100005

**Published:** 2008-01-03

**Authors:** Marcelo Luis Steiner, César Eduardo Fernandes, Rodolfo Strufaldi, Lucia Helena de Azevedo, Cristina Stephan, Luciano Melo Pompei, Sérgio Peixoto

**Keywords:** Osteoporosis, Bone mineral density, Screening, Ultrasonography, Risk factors, Osteoporose, Densidade mineral óssea, Peneiramento, Ultra-sonografia, Fatores de risco

## Abstract

**CONTEXT AND OBJECTIVE:**

Osteoporosis is the greatest cause of quality-of-life reductions, morbidity and mortality among postmenopausal women, with growing incidence as populations age. Clinical tools like Osteorisk provide an easy-access and low-cost alternative method that helps physicians to reduce the need for dual-energy X-ray absorptiometry (DXA), the expensive gold standard examination for diagnosing osteoporosis. The aim here was to study the accuracy of Osteorisk using heel ultrasonography for bone mineral density (BMD).

**DESIGN AND SETTING:**

Cross-sectional study, at Faculdade de Medicina do ABC.

**METHODS:**

A structured questionnaire was applied to 615 postmenopausal women, with anthropometric measurements, Osteorisk calculations and quantitative ultrasound on the heel using Sonost 2000 equipment.

**RESULTS:**

461 women were included, with mean age 60 ± 9 years, weight 67.6 ± 12.9 kg and body mass index (BMI) 28.8 ± 5.0 kg/m^2^. Their Osteorisk classifications were: 61.0% low-risk, 28.4% medium-risk and 10.6% high-risk. Quantitative ultrasound showed 81.3% low-risk, 10.0% medium-risk and 8.7% high-risk regarding osteoporosis. Statistically significant results were observed (p < 0.001) when Osteorisk was correlated with age, years since menopause and BMI. Correlating these same variables with quantitative ultrasound, statistically significant results were observed for age (p < 0.001), years since menopause (p < 0.001) and BMI (p < 0.006). The sensitivity, specificity, negative predictive value and positive predictive value for Osteorisk were 64%, 6.7%, 89% and 30.6%, respectively.

**CONCLUSION:**

Osteorisk is a valid tool for screening for women at low risk of osteoporosis, making it possible for these women not to have to undergo densitometry.

## RESUMO

**CONTEXTO E OBJETIVO:**

Osteoporose é a principal causa de redução de qualidade de vida, morbidade e mortalidade entre as mulheres no climatério, com aumento na incidência conforme o envelhecimento da população. Ferramentas clínicas como Ostorisk fornecem uma alternativa de acesso fácil e de baixo custo que ajudam o clínico a melhorar a eficácia da solicitação da densitometria óssea, exame padrão ouro, porém caro para o diagnóstico de osteoporose. O objetivo deste artigo foi estudar a acurácia do Ostorisk tendo a ultrassonometria de calcâneo como método de avaliação da densidade mineral óssea.

**TIPO DE ESTUDO E LOCAL:**

Estudo transversal, na Faculdade de Medicina do ABC.

**MÉTODO:**

Um questionário estruturado foi aplicado em 615 mulheres menopausadas, com medidas antropométricas, cálculo do Osteorisk e realização de ultrassonometria quantitativa do calcâneo com o aparelho Sonost 2000.

**RESULTADOS:**

461 mulheres foram incluídas, com uma idade média de 60 ± 9 anos, peso de 67,6 ± 12,9 kg e índice de massa corpórea (IMC) 28,8 ± 5.0 kg/ m^2^. A classificação do Osteorisk para o grupo estudado foi: 61,0% baixo risco, 28,4% médio risco, e 10,6% alto risco. A ultrassonometria de calcâneo mostrou 81,3 % baixo risco, 10,0% médio risco e 8,7% alto risco para osteoporose. Os resultados estatisticamente significantes foram observados (p < 0,001) quando o Osteorisk foi correlacionado com idade, anos de menopausa e índice de massa corpórea (IMC). Correlacionando essas mesmas variedades com a ultra-sonometria, resultados estatisticamente significantes foram observados para idade (p < 0,001), anos de menopausa (p < 0,001) e IMC (p < 0,006). A sensibilidade, especificidade, o valor preditivo negativo e o valor preditivo positivo para o Osteorisk foram 64%, 6,7%, 89% e 30,6%, respectivamente.

**CONCLUSÃO:**

Osteorisk é uma ferramenta válida para o rastreamento de mulheres com baixo risco para desenvolver osteoporose, permitindo que estas não precisem ser submetidas à densitometria óssea.

## INTRODUCTION

Osteoporosis is a syndrome characterized by low bone mass and deterioration of the bone microarchitecture, which results in greater fragility of the skeleton and increased risk of fractures.^1^ It is the most common bone metabolism disease, affecting around 200 million people around the world, and it is the greatest cause of diminished quality of life, morbidity and mortality among postmenopausal women.^1–3^

Estrogen deficiency is responsible for 30 to 50% of the bone loss observed during women's lives.^3^ In Brazil, it has been estimated that 10 million individuals are affected by osteoporosis^4^ and that, because of the greater ages attained by the population, the number of hip fractures among men and women between 50 and 60 years old will increase by 400% by 2050, in comparison with the prevalence in 1950.^4^ This signifies a major public health problem with enormous financial cost relating to caring for this morbid condition.^5–10^ It has become important to achieve early identification of individuals at greater risk of this disease, in order to implement preventive measures.^5–10^

Osteoporosis diagnoses are made from bone mineral density (BMD) measurements. BMD measurements by means of dual X-ray absorptiometry (DXA), also known as bone densitometry, are considered to be the gold standard test for diagnosing this disease.^8^ Such measurements provide high precision in evaluating the mineral density of the axial skeleton and enable precise decisions in relation to the treatment and follow-up for the disease. However, DXA is not widely available within public healthcare, thereby making evaluations on all postmenopausal women logistically impossible and prohibitively expensive.^2,8,11–14^

To minimize such difficulties, new methods for measuring BMD have been developed and made available on the market. These methods include peripheral DXA for forearm and heel measurements, peripheral quantitative computed tomography (QCT) for wrist and tibia studies, radiographic absorptiometry for fingers, and quantitative ultrasound or ultrasonometry (QUS) for heels and other areas.^15^

The availability of technology for evaluating bone mass by using peripheral sites in the skeleton has improved test accessibility because the technology is portable, faster, easier to perform and less costly. And most importantly, these methods can be used for calculating fracture risks both in peripheral and in central sites, with similar performance to central BMD measurements, except for calculations on the risk of hip fractures, which may be more precise with bone densitometry of the hip bone. The BMD of the peripheral skeleton, including the distal radius, phalanx and heel, correlates reasonably well with the density of the axial skeleton (hip and spinal column).^8,12^

To have all postmenopausal women undergo these examinations with the aim of screening them for osteoporosis is impossible and not recommendable, considering that many women do not present any risk of osteoporosis and will not develop the disease.^5,11,13^ On the other hand, Siris et al.^8^ studied 200,160 American women over the age of 50 years who had undergone different peripheral BMD measurement methods and found that half of them did not know that they presented decreased BMD, while 7% presented osteoporosis. This finding is very important, because individuals diagnosed with osteoporosis have 2.74 times greater chance of presenting bone fractures within one year, and those with osteopenia have 1.73 times greater chance.^6,8,9^

The ideal would be to have clinical methods capable of identifying patients who are at greater risk of osteoporosis. These methods have still not been defined, since the guidelines are insufficiently precise for selecting such women.^5^ One alternative would be to establish a way of evaluating clinical risk factors that would identify women with greater likelihood of benefiting from such examinations. If these evaluations correctly differentiated the women who presented bone mass loss or osteoporosis from those with normal densitometry results, the need for bone densitometry examinations could be reduced.^11–13,16^

In the recent literature, there is a proven method for identifying osteoporosis risks that is easy to use and has low cost, called the “Osteorisk” risk assessment tool.^17^ Osteorisk has been validated in Asia, Europe, the United States and Latin America, undergoing adjustments in accordance with each population studied.^4^ According to Sen et al., the sensitivity of this method reaches 94% and the specificity, 45%.^4^ Osteorisk is based on a series of statistical calculations and, using age and body weight variables allocated in a defined table, it allows the risk of osteoporosis to be classified as high, moderate or low. On the basis of these results, doctors can identify patients who are at greater risk of low bone mass and request examinations of higher complexity, and even begin therapy if it is impossible to undertake such examinations.^4,5,11–13^

The aim of Osteorisk is not to diagnose osteoporosis or osteopenia, but to identify women with a greater likelihood of developing low BMD, so that they can be advised to undergo bone densitometry examination. In this manner, the effectiveness of detecting patients with osteoporosis and osteopenia is increased and the wastage of unnecessary examinations is avoided.^4,5,11–13,16^

## OBJECTIVE

In the present study, the objective was to compare BMD measurements obtained by means of quantitative ultrasound on the heel and by means of the Osteorisk method, among a population of postmenopausal women. Our aim was to confirm the applicability of the Osteorisk index to our population, and thus to use it in daily medical practice within our service.

## METHODS

### Location and population studied

This study was granted prior approval by the Research Ethics Committee of Faculdade de Medicina do ABC, and was conducted in the municipality of São Bernardo do Campo, in the Greater São Paulo region, which is in southeastern Brazil. São Bernardo do Campo is a predominantly urban municipality with a population of approximately 700,000 inhabitants.^18^ The study was carried out between September and November 2005.

### Inclusion and exclusion criteria

For inclusion in the study, the women had to be over 50 years old and had to have been postmenopausal for at least six months, with or without climacteric symptoms. They had to have had no utilization within the last six months of medications such as estrogen, progestogen, androgen, aromatase inhibitor, bisphosphonates, calcium, corticoids, estrogen modulators, parathormone, fluorine, anticonvulsants or lithium, or high doses of antacids.

Women aged less than 50 years were excluded, as were those who presented severe bone pains, bone implants or histories of fractures, bone metabolism dysfunction, bone metastases, thyroid abnormalities or liver diseases.

### Interview

The patients were called in to undergo ultrasonometry on the heel by invitation issued by community health agents working for the city authorities of São Bernardo do Campo. A structured questionnaire was applied to the individuals who took up the invitation, and it was filled out on their behalf by doctors or medical students. Anthropometric measurements were also made. All the participants were given explanations regarding the examination, and they signed a statement of free and informed consent. By the end of this recruitment period, 615 patients had been interviewed in the study, but 461 women were included in this study.

Detailed descriptions were obtained of any previous diseases among these patients, such as thyroid abnormalities, parathyroid abnormalities, diabetes mellitus, systemic arterial hypertension, rheumatoid arthritis, liver disease and intestinal malabsorption syndromes (neoplasias or gastrointestinal surgery causing reduced absorption). Likewise, details were obtained regarding previous or present use of corticosteroids, thyroid hormones and hormonal therapy.

Information was collected from each individual regarding personal or family histories of bone fractures and osteoporosis, socioeconomic level, habits such as smoking and alcohol use, profession, schooling level and physical activity. The interview also included obtaining information regarding age, menarche, menopause and obstetric and surgical histories.

### Anthropometric measurements

Weight and height were measured using an anthropometric balance, with the patients in orthostatic position, wearing light clothes and without shoes. The body mass index was calculated by means of dividing the weight in kilograms by the square of the height in meters.

### Osteorisk

Osteorisk is an index developed by Sen et al.^4^ to categorize the risk of osteoporosis as low, medium or high. It was constructed from a study carried out in six centers in Latin America. After multivariate regression analysis of eight osteoporosis risk factors, a model using only age and body weight was obtained. Based on this model, Osteorisk is calculated as 0.2 x [(body weight in kg) – (age in years)]. In the low-risk category, the Osteorisk index is greater than 1, while in the high-risk category it is less than −2 and in the medium-risk category it is greater than −2 and less than 1. The results are represented graphically to simplify their clinical use (not shown).^4^

### Measurement of bone mass

Bone mass was measured from the sound velocity (meters per second), by means of ultrasonometry on the heel, using the Sonost 2000 equipment (United States, 2000). This portable apparatus uses gel as the conduction medium and is capable of measuring sound velocities in bones.

The measurement was done on the right heel for all patients. The examinations were performed by a single trained professional. The apparatus was calibrated daily.

The T-score for each individual was calculated using the peak value for sound velocities for a given population of young adults, with standard deviation, by means of the following equation:^19^ T-score = sound velocity (individual) – sound velocity (peak value for young adults)/standard deviation (peak value for young adults). The peak value for the sound velocities for young adults were calculated from an estimated peak bone mass, which was defined as the mean for the maximum bone mass attained by young healthy adults, matched for race and sex. The individual under examination was classified in the following manner:

Low risk: T-score higher than −1.5.
Medium risk: T-score between −1.5 and −1.99.
High risk: T-score lower than −2.0.

To evaluate the sensitivity, specificity, positive predictive value (PPV) and negative predictive value (NPV), the medium and high-risk results were grouped and considered together.

### Statistical analysis

The data were summarized as means ± standard deviations in the case of quantitative variables, and as numbers and percentages for qualitative variables.

The concordance between the ultrasonometry and Osteorisk results was evaluated by means of calculating Kappa statistics accompanied by their respective 95% confidence intervals (95% CI).

The chi-squared test was utilized for evaluating associations between qualitative variables, and analysis of variance (ANOVA) for comparing the means of quantitative variables according to the Osteorisk or ultrasonometry categories. The Brown-Forsythe correction was used in cases in which the equality of variance test was rejected.^20^

For all the statistical analyses, a significance level of 5% (α = 0.05) was adopted. In other words, results that presented p-values less than 0.05 (p < 0.05) were considered to be significant.

The population sample size was calculated by assuming an osteoporosis prevalence of 14.7%,^21^ from which a sample of 374 women was found to be necessary for a precision of 0.035.

### RESULTS

The number of women interviewed was 615. Of these, 154 did not fulfill the inclusion criteria or were within the exclusion criteria. Thus, the final sample was composed of 461 women. Their mean age (± standard deviation) was 60 ± 9 year (minimum = 45 and maximum = 90 years). Their mean weight was 67.6 ± 12.9 kg (minimum = 36 and maximum = 116 kg). Their body mass index (BMI) ranged from 15.8 to 53 kg/m^2^ and the mean BMI was 28.8 ± 5.0 kg/m^2^.

**Figure 1:**
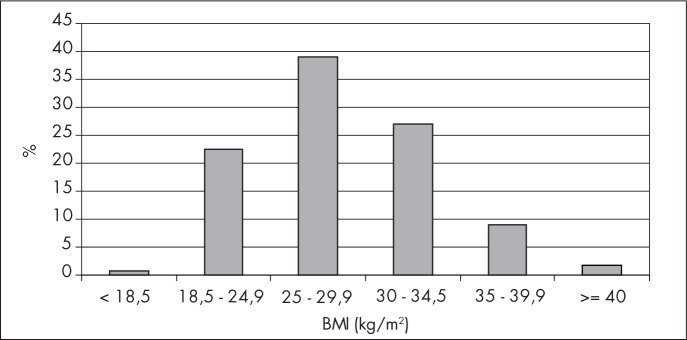
Patient distribution according to body mass index (BMI) classification.

Figure 1 presents the distribution of the 461 women who took part in the study, according to their BMI classification. It can be seen that 180 (39%) of them were classified as overweight (BMI between 25 and 29.9 kg/m^2^) and only three women (0.7%) were underweight (BMI < 18.5).

The Osteorisk classification showed that 61% of the patients presented low risk, 28.4% medium risk and 10.6% high risk of osteoporosis, while ultrasonometry classified 81.3% as low risk, 10% as medium risk and 8.7% as high risk (Tables 1 and 2).

**Table 1.** Patient distribution according to quantitative ultrasound classification

Quantitative ultrasound classification	n (%)
Low risk	375 (81.3%)
Medium risk	46 (10.0%)
High risk	40 (8.7%)
**Total**	**461 (100.0%)**

**Table 2.** Patient distribution according to Osteorisk classification

Osteorisk	n (%)
Low risk	281 (61%)
Medium risk	131 (28.4%)
High risk	49 (10.6%)
**Total**	**461 (100%)**

Considering the ultrasonometry and Osteorisk results together, it was seen that 54.2% of the patients were classified as low risk, 4.6% as medium risk and 3.0% as high risk by both Osteorisk and ultrasonometry (Table 3).

**Table 3.** Patient distribution according to results obtained from Osteorisk and quantitative ultrasound on the heel

Quantitative ultrasound				
Osteorisk	Low risk	Medium risk	High risk	Total (Osteorisk)
Low risk	250 (54.2%)	20 (4.3%)	11 (2.4%)	281 (61.0%)
Medium risk	95 (20.6%)	21 (4.6%)	15 (3.3%)	131 (28.4%)
High risk	30 (6.5%)	5 (1.1%)	14 (3.0%)	49 (10.6%)
**Total (quantitative ultrasound)**	**375 (81.3%)**	**46 (10.0%)**	**40 (8.7%)**	**461 (100.0%)**

### Analysis of Osteorisk in relation to age, BMI and years since menopause.

For Osteorisk, statistically significant results were found in relation to analyses of age, BMI and years since menopause (Table 4). With regard to age, the high-risk patients presented a mean age that was statistically greater than the ages presented by medium and low-risk patients. The medium-risk patients presented a greater mean age than that of the low-risk patients. For BMI, the high-risk patients presented a BMI that was statistically lower than the BMIs of the medium and low-risk patients. Regarding the number of years since the menopause, the high-risk patients presented a statistically greater number of years than did the medium and low-risk patients (Table 4).

**Table 4.** Age, body mass index and years since menopause (mean ± standard deviation) according to Osteorisk

	Osteorisk			p-value*
	Low risk (n = 281)	Medium risk (n = 131)	High risk (n = 49)	
Age (years)	56.2 ± 6.3	62.6 ± 8.4	74.4 ± 8.5	< 0.001
Body mass index (kg/m^2^)	30.9 ± 4.7	25.9 ± 3.4	24.0 ± 3.3	< 0.001
Years since menopause	8.4 ± 7.3	14.1 ± 9.4	26.1 ± 9.8	< 0.001

* Analysis of variance (ANOVA) using Brown-Forsythe correction.

### Analysis of ultrasonometry in relation to age, BMI and years since menopause

It can be seen from Table 5 that the ultrasonometry showed significant differences with regard to the variables of age (p < 0.001), BMI (p = 0.006) and years since menopause (p < 0.001). Thus, patients with osteoporosis presented on average a statistically greater age than did the patients with osteopenia or a normal examination. The BMI statistically differentiated the normal patients from those with osteopenia and osteoporosis, although it was not capable of differentiating between the patients in the latter two groups. The number of years since menopause was statistically greater among the women with osteoporosis than among those with osteopenia and normal results.

**Table 5.** Age, body mass index and years since menopause (mean ± standard deviation) according to quantitative ultrasound

	Quantitative ultrasound			p-value*
	Low risk (n = 375)	Medium risk (n = 46)	High risk (n = 40)	
Age (years)	58.7 ± 8.3	61.4 ± 8.8	70.6 ± 10.2	< 0.001
Body mass index (kg/m^2^)	29.1 ± 4.9	27.3 ± 4.5	26.9 ± 6.0	0.006
Years since menopause	10.5 ± 9.1	15.2 ± 10.1	21.4 ± 11.6	< 0.001

* Analysis of variance (ANOVA) using Brown-Forsythe correction.

### Sensitivity, specificity, positive predictive value and negative predictive value for Osteorisk

It can be seen from Tables 6 and 7 that Osteorisk had sensitivity of 64% for identifying the women who were at low risk and specificity of 66.7% for identifying the women who were at medium and high risk of osteoporosis, taking ultrasonometry as the diagnostic reference. This produced a negative predictive value of 89% and a positive predictive value of 30.6%.

**Table 6.** Correlation between Osteorisk and qualitative ultrasound, considering low risk versus medium and high risk

Osteorisk	Quantitative ultrasound		Total
	Medium and high risk	Low risk	
Medium and high risk	55 (11.9%)	125 (27.1%)	180 (39.0%)
Low risk	31 (6.7%)	250 (54.2%)	281 (61.0%)
**Total**	**86 (18.7%)**	**375 (81.3%)**	**461 (100.0%)**

**Table 7.** Sensitivity, specificity, positive predictive value and negative predictive value for Osteorisk

	Value	95% confidence interval
Sensitivity	64%	[53.4%; 73.3%]
Specificity	66.7%	[61.7%; 71.2%]
Negative predictive value	89.0%	[84.8%; 92.1%]
Positive predictive value	30.6%	[24.3%; 37.6%]

## DISCUSSION

Early identification of patients who are at risk of osteoporosis is of great importance following the menopause. Preventive action by doctors signifies decreased morbidity-mortality due to this disease and decreased public health cost.

Bone densitometry is recognized as the principal tool for diagnosing osteoporosis. The literature demonstrates that when this examination is available for osteoporosis prevention, this has an effect on the number of future fractures.^6–9^ Kern et al.^22^ showed that when bone densitometry was used as a screening method for osteoporosis, there were 36% fewer fractures over a six-year period than when other medical care was utilized. However, indiscriminate screening of all patients by this method is impossible in Brazil because of its high cost and low availability, particularly for the social classes with lower income.

Today, there are methods like ultrasonometry on the heel that can be used for evaluating the risk of fracture, both at peripheral and at central sites, with performance resembling measurements of central BMD. The exception to this is calculations of the risk of hip fractures, which are more precise with bone densitometry on the hip. The BMD of the peripheral skeleton, including the distal radius, phalanx and heel, correlates reasonably well with the density of the axial skeleton (hip and spinal column, and the costs of obtaining these data are more accessible.^2,8,23,24^

In this light, we undertook a comparison between Osteorisk and ultrasonometry on the heel, among women living in São Bernardo do Campo, to evaluate the effectiveness of Osteorisk as a screening method for identifying the patients who were more susceptible to osteoporosis. We evaluated our population in relation to age, BMI and number of years since the menopause. In both the Osteorisk and the ultrasonometry analyses, all these variables presented statistical significance.

BMI has an inverse relationship with osteoporosis, and this was confirmed in our sample, both for Osteorisk and for ultrasonometry. In both analyses, we observed that the low-risk patients had BMI that was statistically significantly greater than among those at high risk.

Age has a direct relationship with osteoporosis, and this was confirmed in our study: low-risk patients as assessed by Osteorisk presented a mean age of 18.2 years lower than did the high-risk patients; and for ultrasonometry on the heel, 11.9 years. In relation to the number of years since the menopause too, our data were concordant with the literature, establishing a directly proportional relationship between the number of years since the menopause and osteoporosis. Low-risk patients as assessed by Osteorisk presented 17.7 years less since the menopause than did those at high risk, and for ultrasonometry this value was 10.9 years.

If Osteorisk were to be proven satisfactory, it could be utilized as an alternative to screening by means of bone densitometry. According to Sen et al.,^4^ Osteorisk presented sensitivity greater than 90% for identifying patients who were at risk of osteoporosis. We applied Osteorisk to the population studied and compared it with the results from quantitative ultrasonometry on the heel, while maintaining the definitions of the World Health Organization.^25^ for osteoporosis, osteopenia and normal bone mass. These were established by measuring bone mass using bone densitometry and comparing this with a young population (T-score).^2,19^

One important matter that is still controversial is the precision of using peripheral measurements for evaluating bone mass, in comparison with the central measurements that are used by WHO.^2,14,22,23^ It has been observed that, in using peripheral measurements (among which ultrasonometry on the heel), there is a lower percentage of osteoporosis identification than when BMD measurements on the axial skeleton are used.^2,6,22,23^ There are references in the literature that defend and utilize a higher cutoff point of −1.8 standard deviations for identifying the risk of osteoporosis, when the BMD is evaluated by ultrasonometry. Through this, the percentage of osteoporosis identification would be very similar to what is found by conventional densitometry.^2,6,22,23^ On the basis of this analysis, we chose in our study to take the women who were at high risk of osteoporosis to be those whose ultrasonometry values were lower than a T-score of −2.0, medium risk to be between −1.5 and −1.99 and low risk to be a T-score higher than −1.5. From this classification, ultrasonometry detected that 8.7% of the women were at high risk of osteoporosis. It should be emphasized that this value was close to the prevalence of osteoporosis identified in the literature by using bone densitometry, which ranges from 7 to 8%.^2,22,23^

We considered it to be of interest to evaluate the validation of the Osteorisk method by identifying the risk of osteoporosis in comparison with ultrasonometry. The values obtained demonstrated sensitivity of 64% and specificity of 66.7% for identifying patients at medium and high risk of osteoporosis. The positive predictive value, which indicates the likelihood that medium and high-risk patients as assessed by Osteorisk are really medium and high-risk using ultrasonometry, was 30.6%. The negative predictive value, which indicates the likelihood that low-risk patients as assessed by Osteorisk are really low-risk using ultrasonometry, was 89%.

Assessing the results, we noted that Osteorisk presented a false negative rate of 36%, in comparison with ultrasonometry. This represents a limitation to the use of this clinical tool, because such patients would fail to be diagnosed, since they would be erroneously considered to be at low risk. It would be of interest to have greater sensitivity in order to use Osteorisk with assurance. Nonetheless, it must not be neglected that Osteorisk also presented a high negative predictive value and would correctly select 64% of the patients who were identified by means of dual X-ray absorptiometry (DXA).

We are aware that our conclusions leave room for criticism. In our defense, we must first cite the fact that our study population included individuals of black race and thus differed from the population evaluated by the authors of the Osteorisk method, which did not include such individuals.^4^ It is known that ethnic origin has a relationship with osteoporosis, which is more prevalent among Caucasians and less so among Africans.^4,8,14^ We can also cite the fact that 77% of the women in our population had a BMI of more than 25 kg/m^2^. This is highly relevant for the sample, since one of the variables for Osteorisk is precisely the weight.

We are also aware that ultrasonometry on the heel is not a gold standard for evaluating BMD and that its real effectiveness in identifying osteoporosis is not totally defined.^2,8,14,16,22,23^ Studies have highlighted its usefulness for identifying patients who are at risk of fractures, but even this idea is not fully grounded. In the national osteoporosis risk assessment (NORA) study, peripheral measurements on patients who presented fractures after 12 months of follow-up showed that only 18% of these patients would have received treatment for fractures if the cutoff criterion had been T-score ≤ 2.5, and 22.6% using the criterion of the National Osteoporosis Foundation (NOF).^8^ Perhaps alteration of the evaluation cutoff point for peripheral measurements, as already described, would be a viable solution that would increase the effectiveness of these measurements for identifying osteoporosis and risks of fractures. Studies in Latin America, and especially in Brazil, comparing Osteorisk and ultrasonometry on the heel with bone densitometry would be of great importance for validating these diagnostic methods.

## CONCLUSION

Our study allows the conclusion that Osteorisk is a valid tool for screening for women who are at low risk of osteoporosis, thereby making it possible not to have to perform bone densitometry on this group.
